# Case Report: Mixed Cholestatic/Hepatocellular Liver Injury Induced by the Herbicide Quizalofop-p-ethyl

**DOI:** 10.1289/ehp.9968

**Published:** 2007-08-24

**Authors:** Ioannis S. Elefsiniotis, George D. Liatsos, Dimitris Stamelakis, Antonios Moulakakis

**Affiliations:** Department of Internal Medicine, Hepatology Unit, “Hippokration” General Hospital, Athens, Greece

**Keywords:** autoimmune drug-induced liver injury, cholestatic/hepatocellular liver injury, drug-induced liver injury, herbicide, hepatotoxicity, quizalofop-p-ethyl

## Abstract

**Context:**

Quizalofop-p-ethyl is an often applied, slightly toxic herbicide for which no severe toxicity has been reported in humans.

**Case presentation:**

We present the case of a farmer exposed to quizalofop-p-ethyl who presented with obstructive cholestasis. A complete workup disclosed no other cause of liver pathology, but liver biopsy established drug-induced hepatotoxicity. The patient was treated with ursodeoxycholic acid and prednisolone, and was recovered fully 70 days after his exposure to the herbicide. The patient was followed for the next 9 months.

**Conclusion:**

Quizalofop-p-ethyl can induce a mixed cholestatic/hepatocellular liver injury. We discuss possible mechanisms implicated in liver injury after exposure to quizalofop-p-ethyl.

**Relevance to clinical or professional practice:**

In patients presenting with mixed cholestatic/ hepatocellular liver injury, occupational exposure to quizalofop-p-ethyl in the course of agricultural use should be investigated.

Quizalofop-p-ethyl {(R)-2-[4-(6-chloro-quinoxalin-2-yloxy)phenoxy] propionic acid; QpE} is a selective, postemergence phenoxy herbicide that belongs to the chemical group of aryloxyphenoxypropionic acids. The aryloxyphenoxypropionic acids, together with the cyclohexanediones class, account for approximately 10% of the global herbicide market (Harwood 1999). QpE is a slightly toxic compound [U.S. Environmental Protection Agency (EPA) toxicity class III] and is irritating to the skin and only slightly irritating to the eyes (U.S. EPA. 1998). We present the first reported case in humans of a prolonged cholestatic/hepatocellular toxicity induced by occupational exposure to QpE.

## Case Presentation

A 75-year-old male farmer presented with a 4-day history of jaundice, with no pain, no associated symptoms of pruritus or fever, and no dark urine or light-colored stools. Physical examination demonstrated jaundice on the sclera (whites of the eyes) and skin, but signs of liver dysfunction or failure were absent. The gallbladder and liver were not palpable, and there was no tenderness or discomfort in the right upper quadrant of the abdomen. No notable findings were present upon examination of the other systems. The patient’s medical history revealed mild chronic obstructive pulmonary disease; the patient was an ex-smoker and had been treated occasionally with inhaled bronchodilators. There was no previous history of biliary tract disease, alcohol abuse, drug addiction, or blood transfusions. The patient reported that he had used QpE to spray his crops 9 days before admission, and he was exposed to a large amount of the chemical.

**Figure 1:**
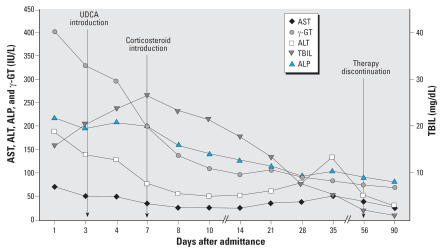
QpE-induced hepatotoxicity shown by a time course of liver biochemistry results for AST, ALT, ALP, γ-GT, and TBIL.

The predominant laboratory findings included markedly elevated serum levels of total [15.85 mg/dL; upper limit of normal range (ULN) = 1.0] and direct bilirubin (13.84 mg/dL; ULN = 0.3), a 2-fold increase in alkaline phosphatase (ALP; 217 IU/L; ULN = 130), and a 5-fold increase in γ-glutamyltransferase (γ-GT; 402 IU/L; ULN = 75). Serum levels of alanine (ALT) and aspartate aminotransferase (AST) were elevated 5-fold (187 IU/L; ULN = 40), and 2-fold (71 IU/L; ULN = 40), respectively (Figure 1). Complete blood count, hematocrit, platelets, serum amylase, albumin and prothrombin time, as well as assessment of kidney function, were all within reference values. Serologic testing for hepatitis (A, B, and C), cytomegalovirus, Epstein-Barr virus, herpes simplex, and varicella zoster was negative. Diagnostic imaging was also performed: Ultrasound of the upper abdomen did not identify any stones or sludge in the gallbladder, or dilations of the intra- and extrahepatic bile ducts. Liver and spleen size were measured within normal range.

During hospitalization the patient remained stable, and at no time did he develop symptoms or signs of liver failure or decompensation. Nevertheless, his serum bilirubin followed an ascending course, peaking at the end of the first week (26.51 mg/dL), whereas ALP, γ-GT, AST, and ALT levels decreased. The patient underwent endoscopic retrograde cholangiopancreatography (catheterization of Vater ampulla was unfeasible because of its ectopic location toward the third segment of the duodenum); subsequently a magnetic resonance cholangiopancreatogram, displayed normal gallbladder, biliary tract, and pancreatic duct anatomy. Further, clinical chemistry for α-fetoprotein and cancer antigens was normal. Electrophoresis of serum proteins, and quantitation and immunofixation assays of immunoglobulins revealed no pathology. Serum testing for antinuclear antibody, smooth muscle antibody, anti-liver–kidney microsome (LKM) antibody, and anti-mitochondrial antibody was negative. In contrast, cytoplasmic antineutrophil cytoplasmic antibodies (c-ANCA) and elevated serum levels of β_2_-microglobulin (β_2_-MG = 4.66 mg/L; ULN = 3.21) were detected.

**Figure 2:**
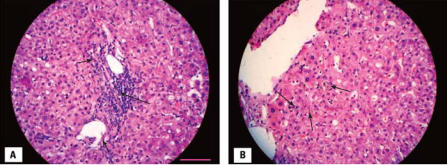
QpE-induced hepatotoxicity indicated by (*A*) Moderate portal inflammatory infiltrates consisting of neutrophils, lymphocytes (long arrow), and eosinophils (short arrows), and (*B*) moderate intralobular cholestasis (arrows). Slides are stained with hematoxylin and eosin. Bar = 100 μm and applies to (*A*) and (*B*).

The patient was treated with ursodeoxycholic acid (UDCA) *per os* (750 mg/day) from the first day of hospitalization. Prednisolone (intravenous dose of 37.5 mg/day) was started after magnetic resonance cholangiopancreatography, with the most prominent diagnosis being cholestatic/hepatocellular drug hepatotoxicity. Finally, after the biliary tree was assessed to have no signs of obstruction, a percutaneous liver biopsy was performed. Moderate portal inflammatory infiltrates consisting of lymphocytes, polymorphonuclear cells, and eosinophils (Figure 2A) plus moderate cholestasis (Figure 2B), mild inflammation, and foci of necrosis in the liver lobule were the hallmarks of the histopathologic liver lesions, which is consistent with drug-induced hepatotoxicity. Two weeks after his admission, the patient was discharged with decreasing serum levels of biliburin (although levels were still high) and continued treatment with UDCA and prednisolone (30 mg/day; followed by a steroid taper). Complete clinical and liver chemistry recovery (Figure 1) was performed 2 months later, almost concomitant with treatment interruption. Nine months after exposure, no further recurrences had occurred.

## Discussion

QpE acts by disrupting acyl lipid biosynthesis via specific inhibition of the plastid isoform of acetyl-coenzyme A carboxylase (ACCase) in grasses. This enzyme catalyzes the first committed reaction in fatty acid biosynthesis, which is virtually ubiquitous in nature. However, as noted by Price et al. (2003), “herbicide-binding cooperativity to the ACCase is the only kinetic property that differentiates naturally” or in “selected insensitive ACCases [found in other monocotyledons, dicotyledons, or other species such as bacteria and animals] from the typical sensitive [isoform] found in grasses.”

According to the U.S. EPA (1998), the acute oral toxicity (lethal dose) of QpE in rats is estimated at 1,480 and 1,670 mg/kg for females and males, respectively; dermal toxicity is > 5,000 mg/kg, and inhalation toxicity (lethal concentration) is 5.8 mg/L. In a 90-day feeding study in mice with diets that contained 0, 100, 316, or 1,000 ppm (approximately 0, 15, 47.4, and 150 mg/kg/ day, respectively), the no observed effect level (NOEL) was < 15 mg/kg/day (the lowest dose tested) on the basis of increased liver weight and reversible histopathologic effects in the liver (centrilobular and generalized hepatocyte enlargement and eosinophilic changes in animals’ livers) (U.S. EPA 1998).

Toxicity for both short and intermediate terms was assessed in a 21-day dermal toxicity study in which New Zealand White rabbits received 15 dermal applications of QpE ester (aqueous paste) at 0, 125, 600, or 2,000 mg/kg/day, 6 hr/day, 5 days/week for 3 weeks (U.S. EPA. 1998). No dermal or systemic toxicity was found; the NOEL was 2,000 mg/kg/day. The U.S. EPA (1998) also found no maternal or developmental toxicity after *in utero* exposure in rats or rabbits.

Neither Mexico nor Canada has maximum residue limits (MRLs) for QpE. Compatibility cannot be achieved with the Canadian negligible residue type limit of 0.1 ppm because data supporting U.S. use patterns showed real residues > 0.1 ppm (U.S. EPA 2005). However, the U.S. EPA (2006) recently proposed a limit for residues of QpE of 0.05 ppm in or on barley, flax (seed), and wheat and 2.0 ppm in or on sun-flower (seed).

It is noteworthy that our literature search found no reports of serious adverse effects on humans [data obtained from the National Library of Medicine (NLM 2006), PubMed (2007), Toxnet (2006), and the EXtension TOXicology NETwork (EXTOXNET 2007)].

The patient mentioned that he had sprayed his crops with QpE 5 months before his admission without presenting any symptoms; however, he used adequate personal protective equipment to handle the QpE at that time. The use of protective equipment, which limited his exposure to the compound, could explain the absence of symptoms at that time.

The second time, the patient applied a mixture of 1 L of 10.3% QpE solution added to 250 L water. His personal protective equipment consisted of protective eyewear, a mechanical filter respirator, chemical-resistant gloves, long pants, and shoes plus socks, but he wore a short-sleeved shirt. During a gust of wind, a large amount of spray mist came in contact with the skin on his upper extremities and neck. Given the fact that he had never presented symptoms from handling herbicides in the past, he continued his work for approximately an hour before discarding his clothing and washing thoroughly with abundant water and soap. Even though the patient’s description explicitly defined the exposure route and time, the amount of chemical could not be estimated.

The recent exposure to QpE in the absence of other causes of liver disease increased our suspicion of drug-induced liver injury (DILI). The patient met all three Council for International Organizations of Medical Science laboratory criteria for DILI: two determinations of ALT plasma concentrations > 2 ULN, conjugated bilirubin > 2 ULN, and combined increases of AST, ALP, and TBIL with one value > 2 ULN (Benichou 1990). According to these criteria, our patient demonstrated an ALT/ALP ratio of 2.5, which classified as a mixed hepatocellular and cholestatic liver injury; those with isolated ALT > 2 ULN or ALT/ALP ratio > 5 are classified as hepatocellular; cases with an ALT/ALP ratio < 2 are cholestatic, and cases with an ALT/ALP ratio between 2 and 5 are classified as mixed liver injuries (Benichou 1990). Histopathologic features of liver biopsy, mainly the portal inflammatory infiltration by neutrophils, lymphocytes, and eosinophils, as well as intrahepatic cholestasis, established the diagnosis of DILI (Watkins and Seeff 2006).

Lee (2003) outlined six possible mechanisms for DILI, and it is important to note that many of these mechanisms can be involved in a chemical-induced liver injury. Chemicals that can damage the structure and function of the bile canaculi can produce cholestasis. A key component of bile secretion involves a series of ATP-dependant export pumps such as the canalibular bile salt transporter (Cullen 2005); binding of a chemical to these transporter molecules results in the arrest of bile formation or movement within the lumen of the canalicular system (Trauner et al. 1998). Secondary injury can result because bile salts have detergent action that can damage cell membranes and injure biliary epithelium or hepatocytes in areas of cholestasis (Cullen 2005). Another mechanism leading to cholestasis is mediated by drugs that bind to actin filaments, resulting in disruption of the actin filaments situated around the bile canaliculi, thus preventing the normal pulsatile contractions that move bile through the canalicular system to the bile ducts (Cullen 2005). One or more of these mechanisms could possibly explain QpE cholestatic hepatotoxicity.

In our patient there was evidence of a drug-induced immunologic disorder. Serology for positive c-ANCA, elevated levels of β_2_-MG, and histology with eosinophil infiltrates in the hepatic lobule suggest an auto-immune mechanism of QpE hepatotoxicity. Adducts can form between drug metabolites and cellular proteins or nucleic acids and generate neoantigens. Neoantigens may be formed on the cell surface by interactions of chemicals with certain cell membrane receptors, or they may be processed, transported to the cell surface, and presented as antigens (Cullen 2005). Depending on the context of the major histocompatibility complex of antigen presentation, either cellular or humoral immunity can be involved (Lewis 2000).

Injury can occur through direct cellular cytotoxicity and antibody-dependent cellular cytotoxicity. Hepatic injury may be significantly exacerbated by recruitment of inflammatory cells such as neutrophils and activation of sinusoidal lining cells, particularly Kupffer cells (Jaeschke et al. 2002). When primed by tumor necrosis factor-α, or interleukin-1 (IL-1), neutrophils interact with extracellular ANCA, resulting in their degranulation and production of reactive oxygen species that can cause tissue damage. In addition, ANCA-activated neutrophils can adhere to, and kill, endothelial cells *in vitro*, and can be induced to release pro-inflammatory cytokines such as IL-1 and IL-8. ANCA are present in a high percentage of patients with certain systemic vasculitis syndromes, particularly Wegener’s granulomatosis and microscopic polyangiitis (Sneller et al. 2005). Even though anti-LKM and anti-LM are the most developed autoantibodies in autoimmune DILI (Watkins and Seeff 2006), the transient appearance of c-ANCA in our patient could be attributed to the induction of immune mechanisms by QpE.

Human leukocyte antigen (HLA) molecules play a critical role in the host immune response because they are involved in antigen presentation. Specifically, class II antigens present foreign antigens to both CD4^+^ helper-T lymphocytes (Th-1 and Th-2), leading to both humoral and cell-mediated immune responses (Delves and Roitt 2000). Serum β_2_-MG levels have been anticipated to represent the turnover of HLA antigens and are associated with lymphocyte proliferation and activation (Bjerrum et al. 1987). In patients with liver disease and without renal impairment or concomitant hematologic disorders, serum β_2_-MG represents a non-specific marker of lymphocyte activation and proliferation, resulting in a Th-1–mediated immune response (Freni et al. 1997). In our patient, serum β_2_-MG elevation possibly follows the drug-induced immunologic reaction of the host. We postulate that the first minimal exposure to the chemical (5 months before admission) sensitized the patient, whereas the second exposure (9 days before admission) triggered the strong immunologic reaction that resulted in hepatotoxicity. It is noteworthy that c-ANCA were undetected 2, 3, and 9 months after exposure, and serum β_2_-MG levels were normal 9 months after exposure (2.51 mg/L). Also, the second low peak in aminotransferase levels, determined in the 35th week (Figure 1), could be attributed either to a second late onset of QpE hepatotoxicity or to steroid treatment (Topal et al. 2006). The patient was treated with UDCA, as reported by Watkins and Seeff (2006). The steroid was initiated 1 week after admission in an attempt to control the continuously ascending bilirubin levels, even though it is not a recommended medication for immune-mediated liver injury in textbooks of internal medicine (Dienstag and Isselbacher 2005).

## Conclusion

We determined that QpE, an often applied herbicide, is a probable inducer of occupational liver injury, according to the World Health Organization (WHO) definitions of adverse drug reactions (WHO 2006). Causality between the compound and liver injury is “probable” because a rechallenge procedure would be unethical. We also demonstrated the biochemical and histologic patterns of QpE-induced liver injury, its “signature” profile, as the pattern of hepatotoxicity was characterized by Watkins and Seeff (2006).
